# Manual Therapy Intervention in Men With Chronic Pelvic Pain Syndrome or Chronic Prostatitis: An Exploratory Prospective Case-Series

**DOI:** 10.7759/cureus.24481

**Published:** 2022-04-25

**Authors:** Carlos Rabal Conesa, Enrique Cao Avellaneda, Pedro López Cubillana, David Prieto Merino, Alexander Khalus Plish, Antonio Martínez Franco, Alicia López Abad

**Affiliations:** 1 Physiotherapy, Universidad Católica de Murcia, Murcia, ESP; 2 Urology, University Hospital Virgen de la Arrixaca, Murcia, ESP; 3 Statistics, Universidad Católica de Murcia, Murcia, ESP; 4 Osteopathic Medicine, Madrid School of Osteopathy, Madrid, ESP

**Keywords:** patient reported outcome measures, musculoskeletal manipulation, physiotherapy, manual therapy, prostatitis

## Abstract

Purpose

Chronic pelvic pain syndrome (CPPS) is permanent pelvic pain of unknown etiology. Current theories suggest a multifactorial origin for CPPS, including urinary pathologies, psychosocial factors, prostate inflammation, infection, central sensitization of the nervous system, and muscular contractures or fibrosis. As there are no defined treatment protocols for CPPS, a multimodal approach is recommended. The objective of this study was to evaluate the impact of a manual therapy treatment protocol on pain, urinary symptoms, and overall quality of life.

Materials and Methods

Twenty-three men aged 47.36 ± 10.11 years were recruited consecutively by urologists practicing at two hospitals. All men presented prostatic tenderness with no other positive clinical history, urine cultures, or echography studies. Patients underwent six manual therapy sessions (three during the first week and three every two weeks after that) performed by a single osteopath or physiotherapist. The intervention protocol addressed the treatment of muscle structures, fascial mechanics, vascularization, innervation, emotional factors, and the need for information. The questionnaires used to evaluate outcomes included the National Institutes of Health Chronic Prostatitis Symptom Index (NIH-CPSI), the International Prostate Symptoms Score (IPSS), and a Visual Analog Scale (VAS) for pain, and the Hospital Anxiety and Depression Scale (HADS). Data were evaluated using Chi-squared or paired difference tests by an external researcher.

Results

The mean NIH-CPSI scores recorded for our study cohort decreased by 7.69 points (30.92%; *p*<0.0005; 95% CI 4.02-10.52). IPSS measurements decreased by 3.20 points (22.18%; *p*=0.009; 95% CI 1.00-6.09), although the item addressing quality of life decreased by 1.67 points only (31.99%; *p*<0.0005; 95% CI 0.94-2.33). The VAS score also decreased by 2.20 points (38.6%; *p*<0.0005; 95% CI 1.45-2.73). Changes in HADS scores were not statistically significant.

Conclusions

Based on patient responses, this case series revealed that manual therapy improved urinary symptoms, pain, and quality of life.

## Introduction

Chronic pelvic pain syndrome (CPPS) involves ongoing or repetitive pain episodes in the pelvic region. These episodes may be exacerbated in response to prostate palpation and are frequently associated with negative perceptions of cognitive/behavioral, sexual, and intestinal transit functions [1]. Diagnosis is established after confirming at least three previous episodes of chronic pelvic pain in the absence of urinary tract infection or other relevant pathology.

Earlier estimates suggest that 10-14% of men in Europe are currently affected by these symptoms [2,3]. The risk of developing chronic prostatitis increases with age and has long-lasting consequences and a significant impact on quality of life [4]. 

CPPS is a heterogeneous syndrome in which each patient manifests various symptoms with different etiologies and progression trajectories. The concurrence of several different pathophysiologic processes (for example, infection, prostatitis, and muscle spasms, among others) that persist over time can result in local and central sensitization leading to pain and autonomic problems [5], including urinary urgency, bowel dysfunction, and ejaculatory issues.

The most widely accepted classifications of CPPS were included in the proposal by the United States (US) National Institute of Diabetes and Digestive and Kidney Diseases (NIDDK). This proposal stratified patients according to the duration of symptoms and the results of a Meares-Stamey four-glass urine test. Our study includes patients diagnosed with CPPS/type IIIB prostatitis (also known as non-inflammatory nonbacterial CP) with negative bacterial cultures and no inflammatory elements detected on the Meares-Stamey test [5].

Numerous questionnaires have been used to assess CPPS symptoms. The most widely used is the Chronic Prostatitis Symptom Index developed at the US National Institutes of Health (NIH-CPSI)[6]. This questionnaire is completed by the patient and thus provides patient-reported outcome (PRO) measures. This index evaluates the three main symptom domains, including pain (scored 0-21), urinary dysfunction (scored 0-10), and impact on quality of life (scored 0-12), thus resulting in a total score of 0-43 after final tabulation. The International Prostatitis Collaborative Network has accepted the NIH-CPSI as a proper and standard tool for assessing male patients presenting with symptoms of CP/CPPS [7].

The International Prostate Symptom Score (IPSS) questionnaire is used widely in studies that focus on lower urinary tract symptoms and is recommended for those charged with helping and managing patients with this condition [8]. The IPSS is a self-administered questionnaire that includes seven PRO items and facilitates the classification of the intensity of lower urinary tract symptoms as mild (1-7), moderate (8-19), or severe (20-35). Using this questionnaire, improvements of three points or more are considered clinically significant [9]. In addition to the NIH-CPPS and IPPS, which focus on urinary symptoms and quality of life issues, the Hospital Anxiety and Depression Scale (HADS) has also been by various research groups to assess the mental states of patients diagnosed with CPPS [10,11].

Due to the clinical complexity of CPPS and the poor results obtained with monotherapy [12], a multi-dimensional approach has been proposed. Therapeutic decisions are primarily guided by the phenotypic classification proposed by Shoskes et al. [13], known as UPOINT (urinary, psychosocial, organ-specific, infection, neurologic/systemic, and tenderness of the skeletal muscle). This classification-based method has been described in guides to clinical practice published by the European Urology Association [5,14]. Manual therapy may be helpful in this context, as this intervention focuses on the structure and mechanical problems that may emerge and emphasizes that health and proper functioning rely on the overall structural integrity of the body. One of the critical targets of manual therapy is the fascial system, which is the complex system of collagen-containing connective tissues that support the body's integrity. Collagen penetrates and surrounds all organs, muscles, bones, and nerve fibers that support its functional structure.

Manual therapy elicits various responses mediated by the central nervous system, including producing analgesic substances, blocking pain signals, improving tissue vascularization, regulating the vegetative system, and improving the patient's emotional state [15-17]. There are only a few high-quality published studies that address the use of manual therapy. As a group, these studies have focused exclusively on the myofascial aspects of the disorder [18] and address its use in combination with relaxation techniques [19] or via a specific osteopathic approach [20]. The clinical results from these efforts were at least similar or, at times, even better than those achieved via treatments routinely recommended in clinical practice guidelines, including drugs.

Given the need to address the different aspects of CPPS and the promising results obtained in trials using manual therapy, it will be necessary to define a reproducible treatment protocol that can be used in conjunction with other therapies to treat patients diagnosed with this syndrome greater scientific rigor. Thus, the primary objective of this work was to evaluate the efficacy of manual therapy techniques for treating the symptoms associated with CPPS as determined using the NIH-CPSI questionnaire. Secondary objectives included efforts to evaluate patient responses concerning urinary function using the IPPS questionnaire, the anxiety/depression component using the HADS questionnaire, and pain intensity measured with a Visual Analog Scale (VAS).

## Materials and methods

Patients

The findings presented are from an exploratory prospective case series and an interventional study conducted among patients diagnosed with CPPS / type IIIB CP according to the NIDKK classification system and treated with specific manual therapy techniques. Male patients >20 years of age were recruited consecutively between September 2014 and September 2016. All patients were evaluated using the following inclusion criteria: (1) prostate tenderness at the first visit to the clinic, (2) negative urine and semen cultures, and (3) written informed consent from the patient. Patients who presented clinical pathologies that would explain the pain symptoms (e.g., herniated disc, urinary tract infection, rectal pathology, anal fissure, and hemorrhoids, among others) had received physiotherapy specifically designed to treat CPPS during the previous six months were excluded from the study.

Management and outcome

A protocol was drafted that addressed the treatment of muscle structures, fascial mechanics, vascularization, innervation, emotional factors, and the need for information (Table 1). Patients remained on their previously-established drug-based treatment protocols.

**Table 1:** Manual therapy techniques included in the intervention, grouped in terms of the anatomical structures they treat.

INTERVENTION PROTOCOL
ARTICULAR
*Global Technique* of the *pelvis*, fascial correction of sacrum, T12 lift
MUSCULAR AND LIGAMENTARY(Bilaterally)
Psoas pumping, suboccipital inhibition, peritoneal equilibration, manual muscle stimulation (ani elevator, transverse perineal, ischiococcygeus), coccyx and perineum release, ischioanal fossa, myofascial reléase (coccygeus, ani levator, superficial transverse perineal, deep transverse perineal, internal obturator, ischiocavernosus, bulbospongiosus)
VASCULAR SYSTEM / N.VEGETATIVE SYSTEM
The global abdominal hemodynamic maneuver, abdominal aorta, arteries and celiac trunk/plexus, upper mesenteric artery, lower mesenteric artery external iliac artery, internal iliac artery.

On the first visit to the clinic, the urologist provided information about the study. Initial findings were collected together with the written informed consent form. The NIH-CPSI, IPSS, and HADS questionnaires and the VAS were provided to each patient with instructions on their self-completion. During the six visits to follow, treatment was provided by the physiotherapist (weekly sessions for three weeks followed by three sessions every two weeks during the six weeks to follow). During the final three visits (at the end of the initial treatment period and again at six to 12 weeks later), the urologist performed a clinical assessment of the patient and delivered the NIH-CPSI, IPSS, and HADS questionnaires and the VAS on each occasion.

Statistical analysis

Statistical analysis was performed by an external researcher who was not involved in performing the intervention. Descriptive statistics were calculated for each variable using frequency distributions for categorical and binary variables; means and standard deviations for used to evaluate quantitative variables. As a component of our inferential analysis, we estimated 95% confidence intervals (CIs) for the means of each quantitative variable evaluated at each visit. We evaluated changes in the means between visits with paired Student t-tests. Normality checks were performed for all variables. Dichotomous variables were analyzed with contingency tables using Pearson's Chi-squared or Fisher's exact test. Statistically significant results were those with P-values less than 0.0016. Analyses were performed using the SPSS statistical software package (IBM Corp., released 2006 Version 15.0, Armonk, NY, USA)

Changes that were considered clinically relevant were as follows: ≥six points in the NIH-CPSI [21], ≥three points on the IPSS [9], ≥1.5 points in HADS subscales [22], and ≥2 points on the VAS [23]. The study was reviewed and approved by the ethics committee of the Universidad Católica San Antonio de Murcia. The study was planned according to the standards of good clinical practice (European Commission, 2005).

## Results

**Figure 1:**
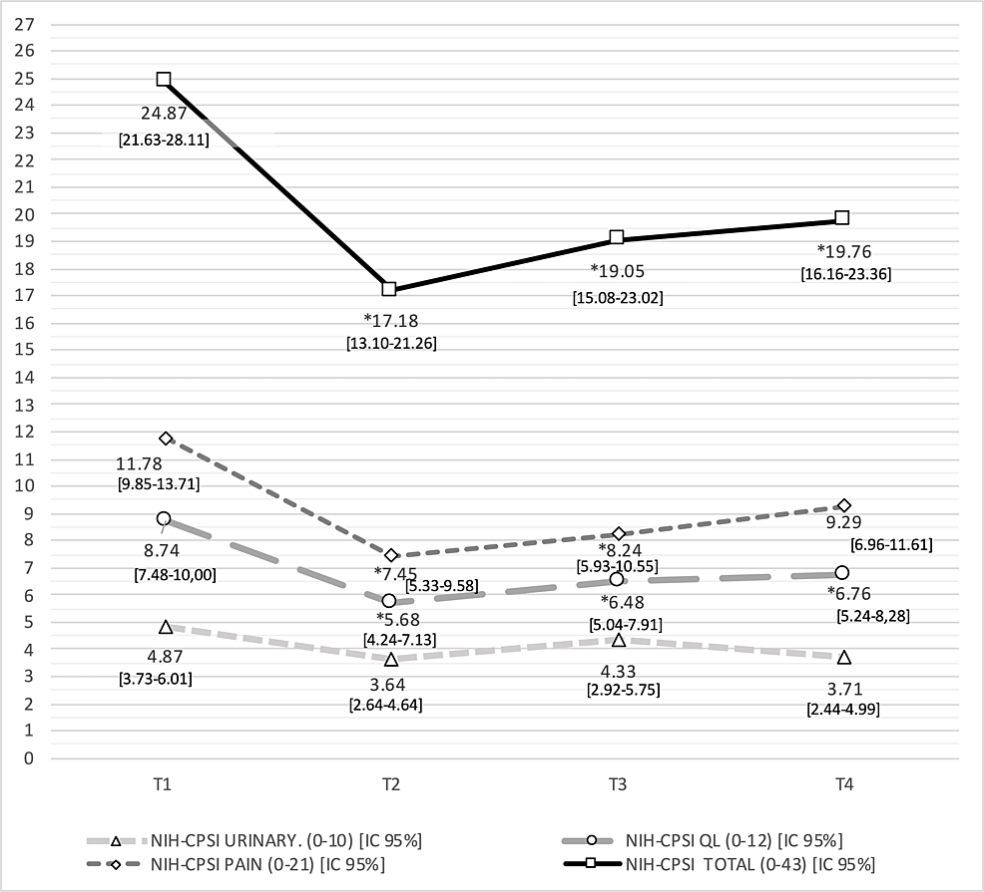
Evolution of mean values obtained in NIH-CPSI questionnaire. Mean values and 95% confidence interval. Results from the NIH-CPSI questionnaire at timepoints T1 through T4. Mean scores and 95% confidence intervals [CIs] are as shown. Abbreviations: NIH-CPSI, National Institutes of Health Chronic Prostatitis Symptom Index; QL, quality of life; T1, baseline pre-intervention evaluation; T2, post-intervention evaluation; T3, evaluation 6 weeks after completion of the intervention; T4, evaluation 12 weeks after completion of the intervention. * P< 0,016.

Fifty-four patients were screened; twenty-three met the inclusion criteria, underwent the intervention, and completed the study (Table 2). The median patient age was 47.35 ± 10.11 years, ranging from 24 to 66 years. Patients reported that they had been diagnosed with type IIIB CPPS on average two years before the study began (range, 1-15 years). The patients reported that they experienced the first symptoms of this disorder on average three years before enrolling in the study (range, 1-15 years). While 78% of the patients were treated with as many as three drugs, 26% were on no medications. At the baseline visit (T1), the mean score on the NIH-CPSI questionnaire was 24.87 (95% CI, 21.63-28.11). When subdivided by domain, the mean pain score was 11.78 (95% CI, 9.85-13.71), the mean score for urinary symptoms was 4.87 (95% CI, 3.73-6.01), and the quality of life (QoL) score was 8.74 (95% CI, 7.48-10.00). The mean baseline (T1) score on the IPSS questionnaire was 14.43 (95% CI, 10.79-18.08); the mean score in the QoL domain was 5.22 (95% CI, 4.81-5.63). The mean baseline (T1) HADS score was 13.92 (95% CI, 10.62-17.21), including mean scores of 6.22 on the anxiety subscale (95% CI, 4.44-7.99) and 7.7 on the depression subscale (95% CI, 5.78-9.61). While the values for anxiety are well within normal limits, those on the depression subscale are comparatively low (Fig. 1). Responses to the VAS for pain resulted in a mean score of 5.70 (range, 2-9].

**Table 2:** Description of the population SD: Standard deviation

PARTICIPANTS 23	MEDIAN (SD)
Age	47,36 (10.11)
Years first diagnostic	2 (3.39)
Years first symptoms	3 (4.07)
EDUCATIONAL LEVEL	N (%)
Basic	8 (34.8)
Medium	6 (26.1)
University	9 (39.1)
EMPLOYMENT SITUATION	N (%)
Unemployed	1 (4.3)
Employee	19 (82.6)
Early retirement, disability or service	2 (8.7)
Retired	1 (4.3)
DRUG CONSUMPTION (%)	N (%)
0	6 (26.1)
1-3	12 (52.1)
>3	5 (21.6)
INCOME	N (%)
Less than 7,404.00€	3 (13)
7,404.74 - 18,510.00€	7 (30.4)
18,510.74 - 37,020.00€	8 (34.8)
37,020.74 - 74,040.00€	5 (21.7)

**Figure 2:**
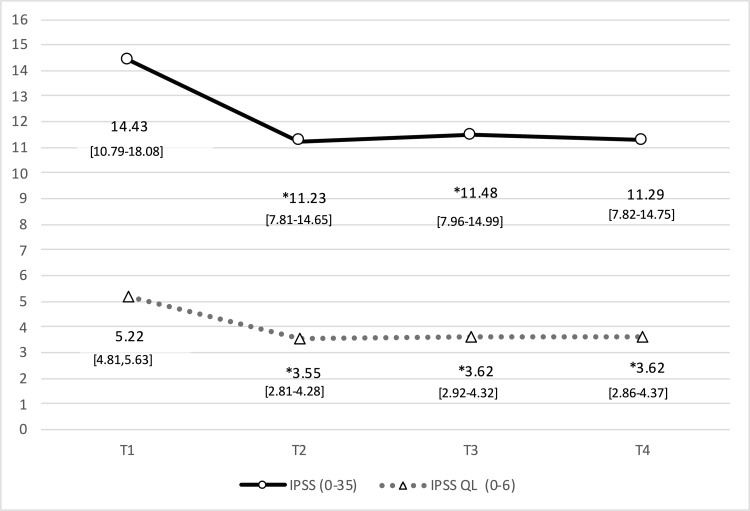
Evolution of mean values obtained with IPSS and IPSS-QoL questionnaire. Mean values and 95% confidence interval. Results from the IPSS and IPSS-QL questionnaires at timepoints T1 through T4. Mean values and 95% [CIs] are as shown. Abbreviations: IPSS, International Prostate Symptom Score; IPSS-QL, International Prostate Symptom Score-Quality of Life; others as in the legend to Figure 1.

**Figure 3:**
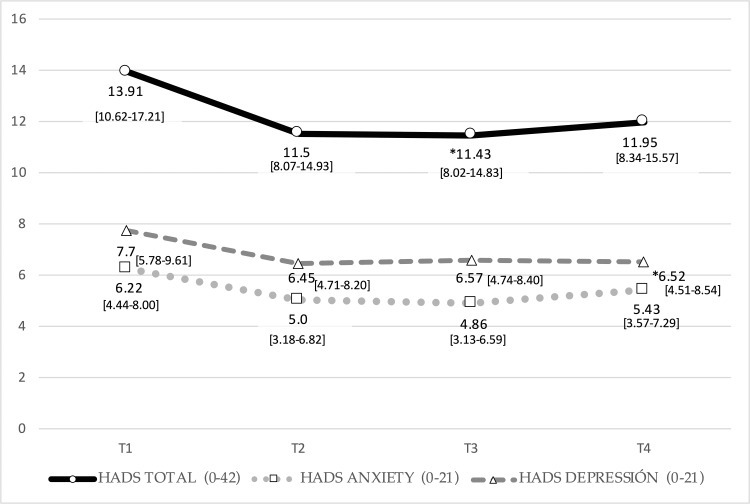
Evolution of HADS scores. Mean values and 95% confidence interval. Results from HADS questionnaires at timepoints T1 through T4. Mean values and 95% [CIs] are as shown. Abbreviations: HADS, Hospital Anxiety and Depression Scale; others as in the legend to Figure 1.

**Figure 4:**
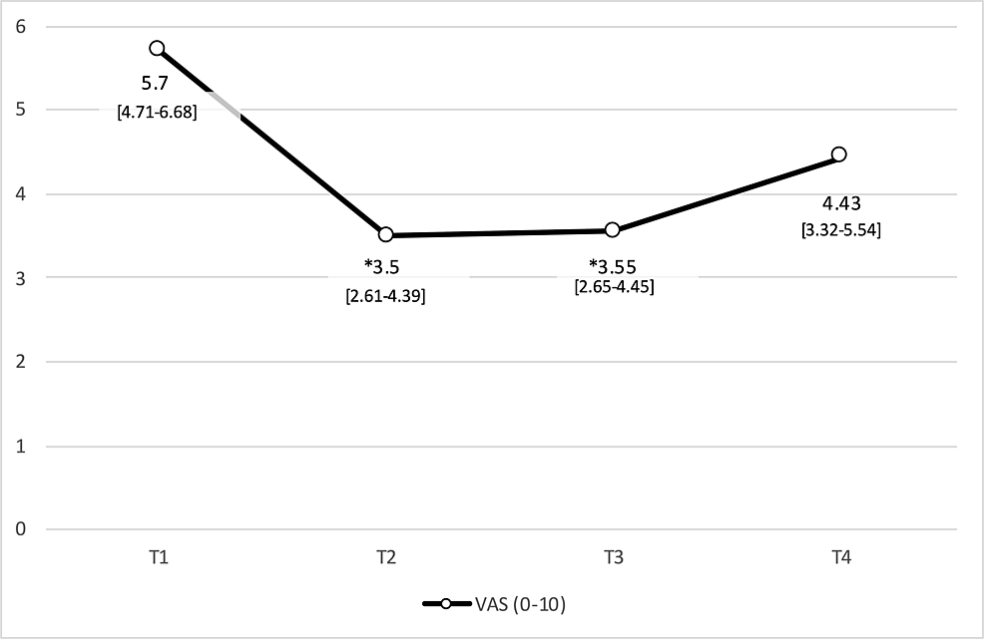
Evolution of mean scores obtained with pain VAS. Mean values and 95% confidence interval. VAS score pain measurements. Mean values and 95% [CIs] are as shown. Abbreviations: VAS, Visual Analog Scale; others as in the legend to Figure 1.

Mean scores and statistical analysis of the results from all the questionnaires and the VAS for pain are shown in (Tables 3, 4), and (figures 1-4). Results include those obtained at baseline (T1) and immediately after completion of the treatment protocol (T2) as well as six (T3) and 12 (T4) weeks after treatment. Our analysis of the results from the NIH-CPSI questionnaire (Fig. 1) revealed a statistically significant decrease of 7.69 points at T2 to scores that were 30.92% lower than those obtained at baseline (p=0.001); a slight increase followed this in scores obtained at T3 and T4. Results from the IPSS (Fig. 2) included a statistically significant decrease in mean values obtained immediately after the intervention (T2; p=0.009), followed by slight increases at six (T3) and 12 weeks (T4) after that. While no significant changes were observed at T2 when using the HADS questionnaire (Fig. 3; p=0.090), significant improvement (p=0.003) was observed at the six-week (T3) but not at the 12-week assessment. These patterns were observed for both the anxiety and depression subscales; statistically significant differences in the depression subscale were only observed at the T4 time point. The findings are shown in Fig. 4 document changes in patient-assessed pain intensity presented as VAS scores. The results revealed both clinical and statistically significant improvements in VAS scores immediately after intervention (T2; p=0.001), with a mean decrease of 38.6%. The mean value increased slightly at six weeks (p=0.000) and again at 12 weeks (p=0.017), although not the final scores did not return to baseline levels.

**Table 3:** Results of NIH-CPSI questionnaire. Abbreviations: NIH-CPSI, National Institute of Health Chronic Prostatitis Symptom Index; NIH-CPSI PAIN, pain subscale; NIH-CPSI URINARY: urinary symptom subscale; NIH-CPSI QoL: Quality of life subscale;  N, number of subjects; T1, baseline pre-intervention evaluation; T2, post-intervention evaluation; T3, evaluation 6 weeks after intervention; T4, evaluation 12 weeks after intervention; [CI 95% ], 95% confidence interval.

	T1-T2		T1-T3		T1-T4	
	T1 -T2	[CI 95%]	T1-T3	[CI 95%]	T1-T4	[CI 95%]
n=23	p-value	%	p-value	%	p-value	%
NIH-CPSI TOTAL (0-43)	7.69	[4.02-10.52]	5.82	[1.80-8.49]	5.11	[1.18-7.68]
	0.000	30.92	0.004	23.40	0.010	20.55
NIH-CPSI PAIN (0-21)	4.33	[2.35-5.74]	3.54	[1.22-4.97]	2.49	[-0.09-4.18]
	0.000	36.76	0.003	30.05	0.059	21.14
NIH-CPSI URINARY (0-10)	1.23	[10.24-2.30]	0.54	[-0.72-1.86]	1.16	[0.05-2.33]
	0.018	25.26	0.367	11.09	0.041	23.82
NIH-CPSI QoL (0-12)	3.06	[1.42-4.40]	2.26	[0.65-3.45]	1.98	[0.51-3.02]
	0.001	35.01	0.006	25.86	0.008	22.65

**Table 4:** Results of IPSS and HADS Questionnaires and VAS. Abbreviations: N, number of subjects; IPSS: International Prostate Symptom Score; HADS: Hospital Anxiety and Depression Scale; VAS: Visual Analogue Scale; [CI95%], 95% confidence interval.

	T1 -T2		T1-T3		T1-T4	
	T1 -T2	[CI 95%]	T1-T3	[CI 95%]	T1-T4	[CI 95%]
n=23	p-value	%	p-value	%	p-value	%
IPSS (0-35)	3.20	[1.00-6.09]	2.95	[0.69-5.31]	3.14	[0.67-5.7]
	0.009	22.18	0.013	20.44	0.016	21.76
IPSS QoL (0-6)	1.67	[0.94-2.33]	1.6	[0.84-2.21]	1.6	[0.84-2.21]
	0.000	31.99	0.000	30.65	0.000	30.65
HADS TOTAL (0-42)	2.41	[-0.36-4.64]	2.48	[0.78-3.21]	1.96	[0.18-2.77]
	0.090	17.33	0.003	17.83	0.027	14.09
HADS ANXIETY (0-21)	1.22	[-0.14-2.32]	1.36	[0.19-2.18]	0.79	[-0.46-1.70]
	0.079	19.61	0.021	21.86	0.247	12.70
HADS DEPRESSION (0-21)	1.25	[-0.49-2.67]	1.13	[-0.15-1.77]	1.18	[0.193-1.52]
	0.167	16.23	0.094	14.68	0.014	15.32
VAS (0-10)	2.20	[1.45-2.73]	2.15	[0.92-2.47]	1.27	[0.20-1.80]
	0.000	38.60	0.000	37.72	0.017	22.28

## Discussion

CPPS involves ongoing or repetitive pain in the pelvic area without infection or other causes that might explain the clinical symptoms. The patient experiences pain on prostatic palpation and frequently develops negative perceptions involving cognitive/behavioral, sexual, and intestinal transit dysfunction. Central sensitization/hypersensitivity phenomena have been implicated in the etiology of this disorder. Although widely studied, there is no strong evidence available that supports specific diagnostic and management protocols. The multimodal theory known as UPOINT described by Shoskes et al. [24], is currently the most widely accepted approach to diagnosis and treatment [25].

This study suggests that manual therapy might be a practical therapeutic approach that provides patients with critical assessments and treatment. The interventions introduced in this trial were provided in six sessions and were administered over nine weeks. The same protocol was applied to all patients to eliminate confounding factors. The intervention was adjusted to meet the study objectives. The techniques employed in the intervention were all manual and external. They aimed to achieve normal alignment of the articular structures, reduce muscle stress, create flexible fascial structures, and improve vascularization and innervation in the affected area. Ongoing active treatment regimens, mainly drug-based, were maintained during the present intervention.

The results from the NIH-CPSI questionnaire revealed an overall improvement of 7.69 points, or 30.92%, from the pre-intervention (T1) to post-intervention evaluations (T2). However, the impact of the treatment protocol was reduced to a mean of 5.82 and 5.11 points at six (T3) and 12 weeks (T4) after treatment. A reduction of six points or a 25% improvement is clinically relevant [26]; the intervention provided in this study may be an effective modality for the treatment of type IIIB CP/CPPS.

Clinical practice guides for CPPS published by the European Association of Urology state that there is stronger support for pharmacologic treatment [5]. Treatment strategies that include antibiotics, α-blockers, or anti-inflammatory agents as monotherapy or in combination are currently supported by the most substantial evidence (1a and 1b). Recently, Franco et al. [12] published a Cochrane Collaboration meta-analysis that compared the impact of various drugs with a placebo. Their results reported improvements in NIH-CPSI scores by 5.01 points with α-blockers, 0.87 points with 5α-reductase inhibitors, 2.43 with antibiotics, 2.5 with anti-inflammatory agents, and 5.02 with phytotherapy. A previous meta-analysis by the same authors [27] evaluated the impact of non-drug-based therapies reported improvements of 5.79 points after acupuncture, 3.90 points in response to lifestyle modifications, 2.50 points after specific (versus non-targeted) physical exercises, 6.18 points after shockwave therapy, 11.2 points in response to stimulation of the tibialis anterior (versus no intervention), and 2.80 points with sono-electromagnetic therapy (compared with placebo).

Very few studies of adequate quality have been published that explore the use of manual therapies for CPPS. Anderson et al. [27] performed an uncontrolled study in which patients underwent intensive therapy for 3-5 hours daily for six days, including manipulation and relaxation techniques and psychotherapy. At six months after therapy, the average reduction in NIH-CPSI scores was 7 points. Similarly, Fitzgerald et al. [19] administered personalized myofascial therapy in a controlled, non-randomized trial that included male and female patients with chronic pelvic pain. The intervention consisted entirely of manual therapies for muscles and connective tissue administered over an average of 10 sessions complemented by exercises to be performed at home. NIH-CPSI questionnaires revealed a mean reduction of 14.4 points (43%) from an initial score of 33.5 points; this included a 6.2-point reduction in pain, 3.9-point reduction in urological symptoms, and 4.2-point change in QoL. By contrast, the Cochrane review published by Franco et al. [28] reported only a one-point reduction in response to myofascial therapy at the final time point compared to the control group. However, the control groups included patients who received non-specific manual therapy in this case. Given that manual therapy can have an overall effect extending beyond the specific treatment area, comparisons to a control group provided with placebo might have resulted in larger effect size. However, even when the same or similar questionnaires are used to assess outcomes, it is difficult to compare the results of different studies, given variations in the extent and severity of the disorder at initial evaluation and the high variability of the interventions performed.

The results obtained in this study are broadly comparable to those reported previously. For example, Marx et al. [20] reported an average 14-point improvement in NIH-CPSI scores in response to manual techniques interventions; however, the lower baseline values reported for the study subjects (average 22.95 points) may provide some explanation for the larger impact observed in this study. Similarly, this group devised personalized treatments for the pelvic area that were administered both internally and externally over five sessions. A similar trial of manual therapy for CPPS published by this group in 2013 [29] revealed improvements of 1.8 and 1.3 points at follow-up that took place 1.5 and 5 years later, respectively. However, the authors did not provide information on the types of treatment that may have been provided during the six weeks and 18 months after completion of the intervention, thus limiting the extent to which our results can be compared to theirs. However, we believe that their results may have surpassed ours because the interventions were adapted specifically for each patient; by contrast, our study featured a closed protocol used for all patients and during all sessions.

Ajimsha et al. [18] published an uncontrolled study that included five sessions of myofascial therapy in the pelvic area provided to 31 men diagnosed with CPPS. This study, which featured manual treatment only, resulted in a 20-point improvement in the total NIH-CPSI score. This represented a 50% reduction from baseline values, a superior response to that achieved with some drug regimens.

The interventions provided in our study resulted in mean reductions of 4.33 (36.76%), 3.54 (30.05%), and 2.49 points (21.14%) at T2, T3, and T4, respectively, in the pain subscale of the NIH-CPSI score. The results were no longer statistically significant at the 12-week evaluation (p=0.059). Similarly, our results revealed initial improvements in the overall and QoL scores of the IPSS questionnaire maintained over the remaining weeks of the study period. The impact of manual therapy both immediately post-intervention (at T2) and at 12 weeks after that (T4) was clinically relevant.

The Hospital Anxiety and Depression Scale (HADS) was also used to assess the outcomes of our study. Our manual therapy protocol resulted in a 2.4-point reduction at T2 from the baseline average score of 13.91 points at T1; this difference did not achieve statistical significance (p=0.090). Improvements in HADS scores of 2.48 and 1.96 points were observed at T3 and T4, respectively. Albeit not statistically significant, these values were higher than the 1.5 points deemed clinically relevant in previous studies [22].

Our intervention also resulted in a reduction in the mean VAS score immediately after the intervention (T2); this effect was lost by the end of the study. As reported by Fortaleza of this studio, we found that PRO questionnaires were a useful method for evaluating outcomes as they eliminated the risk of measurement bias. Multimodal approaches to the treatment of CPPS that include a combination of both manual and instrumental techniques that have already been identified as effective might be combined to produce more powerful and lasting outcomes.

Limitations

This study included several limitations. First, we recognize that the intervention provided in this study included only six sessions. It is possible that prolonging the manual therapy protocol might improve outcomes. Furthermore, an identical protocol was administered to all patients. This may have limited the extent of the improvements observed as the symptoms and origins of this syndrome are heterogeneous and frequently change over time.

Likewise, given the underdiagnosis of this pathology and, by extension, the difficulties associated with enrolling study subjects, no prior sample calculation was made. Instead, we opted to enroll patients consecutively during the intervention period. Despite these limitations, we believe that this study and those similar to it present the basis for future controlled randomized clinical trials that consider the impact of effective multidisciplinary approaches to this clinical condition. Future research studies should include control subjects and larger patient samples to facilitate the collection of more consistent results.

## Conclusions

According to the questionnaires used for the evaluation, manual therapy reduced the pain reported by the patients with CPPS/type IIIb CP who were enrolled in this case series. Manual therapy also resulted in reductions in their urinary symptoms and improved quality of life. These findings are similar to those obtained in other trials of instrumental therapies and response to drug treatment.
